# Effect of the Degree of Substitution on the Hydrophobicity, Crystallinity, and Thermal Properties of Lauroylated Amaranth Starch

**DOI:** 10.3390/polym12112548

**Published:** 2020-10-30

**Authors:** Vicente Espinosa-Solis, Yunia Verónica García-Tejeda, Everth Jimena Leal-Castañeda, Víctor Barrera-Figueroa

**Affiliations:** 1 Coordinación Académica Región Huasteca Sur de la UASLP, Universidad Autónoma de San Luis Potosí, km 5, Carretera Tamazunchale-San Martín, 79960 Tamazunchale, Mexico; vicente.espinosa@uaslp.mx; 2 Instituto Politécnico Nacional, Academia de Ciencias Básicas, UPIITA Avenida Instituto Politécnico Nacional No. 2580, Col. Barrio la Laguna Ticomán, 07340 Gustavo A. Madero, Mexico; 3 Instituto Politécnico Nacional, Academia de Matemáticas, ESIA. 07738, Col. Barrio la Laguna Ticomán, 07340 Gustavo A. Madero, Mexico; elealc@ipn.mx; 4 Instituto Politécnico Nacional, Sección de Estudios de Posgrado e Investigación, UPIITA, Avenida Instituto Politécnico Nacional No. 2580, Col. Barrio la Laguna Ticomán, 07340 Gustavo A. Madero, Mexico; vbarreraf@ipn.mx

**Keywords:** amaranth starch, lauroylation, adsorption isotherms, thermal properties

## Abstract

In this paper, we consider amaranth starch extracted from the seeds of *Amaranthus hypochondriacus* L. An amphiphilic character is conferred to the starch by a chemical modification, which involves an esterification by lauroyl chloride at three modification levels. The degree of substitution (DS) after the modification ranged from 0.06 to 1.16. X-ray photoelectron spectroscopy analysis confirmed the presence of fatty acyl chains on the surface of the esterified starches. The hydrophobicity of starches was confirmed by their adsorption isotherms, which showed a decrease in the moisture adsorption of lauroylated as DS increased. X-ray diffraction analysis revealed a higher crystallinity, which was observed in the two samples subjected to the highest levels of modification. A higher crystallinity is related to a higher gelatinization enthalpy. These results are in agreement with the thermal characterization obtained by differential scanning calorimetry (DSC). An inhibition of the retrogradation properties of lauroylated amaranth starches was also observed.

## 1. Introduction

Amaranth (*Amaranthus* spp.) is considered as an alternative crop to support food security. This crop is extremely adaptable to adverse growing conditions such as drought and low fertility, and it has the potential to well support climate changes [1,2]. Amaranth belongs to the genus *Amaranthus* of the family *Amaranthaceæ* in the order *Caryophyllales*, and it consists of about 75 species [3]. Amaranth seed flour contains approximately 12.5–21% protein, 65–75% starch, 4–5% dietary fiber [4] and 7.1–8.5% oil [5]. In addition, amaranth possesses higher levels of minerals (calcium, magnesium, zinc and iron), vitamins (E, B2 and C) and bioactive compounds than other grains commonly used. Amaranth contributes with 50–102% of the daily requirements of the essential amino acids (histidine, isoleucine, lysine, threonine and valine) for 6–23-month-old children [6], compared to the 15–46% contribution from maize and 20–51% from wheat [7].

Amaranth flour has been investigated as a candidate for substituting wheat gluten in the formulation of the following products: cookies, nuggets and pasta [8,9,10]. Amaranth starch is the main component of amaranth grain, equivalent to 55–70% of its total dry weight [11,12]. Amaranth starch can be classified as an extremely-small-granule starch (0.3–2 μm), as the starches from quinoa, cow cockle and pigweed. Starches can also be classified into large-granule starches (30–100 μm) found in tubers such as potato and canna; medium-granule starches (5–30 μm) found in tapioca, barley, maize and sorghum; and small-granule starches (2–10 μm) found in rice, oat and buckwheat [13].

Chemical modifications of amaranth starch improve its functional properties. Oxidized maize starch with high degree of oxidation can be used to produce thermoplastic starch, which has potential applications as an environmentally-friendly material [14]. Hydroxypropylated amaranth starch is an excellent agent for encapsulating lemon oil [15]. Oxidized amaranth starch is a good substitute for gum arabic in flavor encapsulation [16]. Phosphorylated, acetylated and octenyl succinic anhydride-modified amaranth starches are suitable materials for encapsulation of probiotics [17,18].

The good performance of amaranth starch in the mentioned applications is due to its very small granule size; however, the hydrophilic nature of amaranth starch limits its use in the manufacture of waterproof materials. To overcome this inconvenience, the esterification with saturated fatty acids of medium and long chain is a promising method to confer hydrophobicity, thermoplasticity and amphiphilic properties to starches [19]. The acylation of starch with fatty acid chlorides in aqueous sodium hydroxide solution is an economical method for the preparation of esterified starches: the reaction time is short; the acyl chlorides are hydrolyzed under reaction conditions and converted into sodium chloride; and the starch derivative is precipitated and separated easily [20]. It its worth mentioning that the esterification of starch through the use of fatty acid chlorides allows obtaining high degrees of substitution because no restrictions on the use of fatty acid chlorides exist, unlike other reagents widely used in esterification, such as acetic anhydride and octenyl succinic anhydride (21CFR-172.892).

The esterification of starch by using fatty acid chlorides of short chain (C1–C5) is not recommended, because these competitively react with the water instead of the hydroxyl groups on starch molecules. The reaction of the fatty acid chloride with water produces hydrochloric acid (HCl), which hydrolyzes starch chains [20]. The degree of substitution decreases on increasing the chain length of the fatty acid chloride due to the steric hindrance effect [21].

Lauroyl chloride has been used for the synthesis of lauric arginate, a food-grade cationic surfactant that is accepted as a food-grade ingredient (GRAS). The relatively low toxicity of lauric arginate has been attributed to the fact that it is split into an amino acid and a free fatty acid in the gastrointestinal tract [22]. The esterification of starch with lauroyl chloride has been little studied. Recently, it was used to produce lauroylated amaranth starch for pickering emulsions [23] with a DS = 0.01. To the best of our knowledge, there are no studies regarding the relationship between the DS of lauroylated amaranth starch and its hydrophobicity. It is necessary to carry out more structural studies to promote its use in food and non-food applications. One of these studies is to evaluate the degree of hydrophobicity conferred as the degree of lauroylation increases.

In the present work, the effect of esterification on amaranth starch at three levels of lauroyl chloride is analyzed. It is hypothesized that the higher is the level of esterification on the starch, the higher is the conferred hydrophobicity. Comparative analyses of native starch and lauroylated starches were performed by moisture adsorption isotherms to determine its hydrophobicity by determining the monolayer moisture content from the Guggenheim–Anderson de Boer model (GAB) [24]. The surface characterization of amaranth starch and their derivatives by XPS (X-ray Photoelectron Spectroscopy) is reported. In addition, DSC (Differential Scanning Calorimetry) was used to analyze the thermal properties of starches including gelatinization and retrogradation.

## 2. Materials and Methods

### 2.1. Plant Materials and Chemical Reagents

Seeds of *Amaranthus hypochondriacus* from cultivar Nutrisol were donated by the National Research Institute in Forestry, Agriculture and Livestock, México (INIFAP). Lauroyl chloride was purchased from Sigma-Aldrich Chemical Co. (Toluca de Lerdo, México). The analytical grade salts NaOH, LiCl, CH_3_CO_2_K, KCl, K_2_CO_3_, Mg(NO_3_)_2_, NaCl, KCl, NaHSO_3_ and BaCl were purchased from J. T. Baker^®^ (Phillipsburg, NJ, USA).

### 2.2. Extraction of Amaranth Starch

Extraction of amaranth starch was performed according to the alkaline wet-milling method described in [17]. Amaranth seeds (1kg) were steeped in 2 L of 0.1 mol/L NaOH solution for 24 h. After steeping, the seeds were washed with distilled water and posteriorly milled together with a solution 0.1 mol/L NaHSO_3_ in a stone mill (Fumasa, Querétaro, México). The steeped mass was screened through U.S. standard sieves (420, 250, 177, 149, 74 and 62.5 μm) to collect the steep water, which was then centrifuged at 6000× *g* for 20 min. The supernatant was discarded, and the top yellowish layer of protein was removed with a laboratory spatula. The starch was dried in a convection oven at 40 °C for 48 h. This methodology allowed us to obtain 90.04 ± 0.01% of total starch, which was determined by using the kit from Megazyme International Ireland Ltd. (Bray Business Park, Wicklow, Ireland) based on the use of thermostable α-amylase and amyloglucosidase [25].

### 2.3. Lauroylation of Amaranth Starch

Esterification of amaranth starch was carried out according to the method of Leal-Castaneda et al. [23]: 4 g (25 mmol AGU) of starch were dispersed under mechanical stirring (300 rpm), into 40 mL of NaOH 0.1 M at 25 °C for 10 min, then lauroyl chloride was added to the starch dispersion drop-wisely during 10 min, and the reaction was completed after 10 min. The volumes of lauroyl chloride for the three modification levels were 2.5 mL (10.42 mmol) for ASL1, 5 mL (20.83 mmol) for ASL2 and 7 mL for ASL3 (31.25 mmol). After the esterification reaction, the slurries were centrifuged for 10 min at 1600× *g*, and then the starch laurates were recovered. The starch laurates were washed three times with ethanol and one time with distilled water, and then theu were centrifuged and dried in a convection oven at 40 °C for 24 h. The dried starches were milled in a coffee grinder (Hamilton Beach^®^, Glen Allen, VA, USA, model 80350) and sieved in a mesh 100 of 0.149-mm opening size.

### 2.4. Degree of Substitution

The degree of substitution (DS) was determined by ^1^H nuclear magnetic resonance spectroscopy (^1^H NMR), using a Bruker Avance 750 spectrometer (Bruker Biospin, Karlsruhe, Germany) operating at 750.1 MHz and equipped with a 5 mm TXI probe at 298 K. The starch samples were dissolved in deuterated dimethyl sulfoxide and placed in NMR tubes according to the methodology reported by Leal-Castaneda et al. [23]. ^1^H NMR spectra were obtained using the pulse program 1D NOESY-presat. DS was calculated from the ratio of the signals of the three protons of the terminal methyl group of the acyl chain and the four protons of the anhydrous glucose units, according to the following formula:

$$D=\frac{I_{\mathrm{signal}}/3}{I_{\mathrm{AGU}}/4}$$

where 3 stands for the number of protons from the signal $I_{\mathrm{signal}}$ of the methyl proton and $I_{\mathrm{AGU}}$ is the integral for the 4 protons of the anhydrous glucose unit (AGU for short) between 4.58 and 5.50 ppm [21].

### 2.5. X-ray Photoelectron Spectroscopy (XPS)

The samples were dried in high vacuum ($1.9\times 10^{-8}$–$9.8\times 10^{-9}$ mBar) before being transferred to the analysis chamber. To obtain the general spectra, a scan from 0 to 1370 eV was performed, using a K-Alpha Thermo Scientific (Thermo Fisher Scientific Inc., Loughborough, UK) photoelectron spectrometer, which uses a monochromatic AlK-α source (1487 eV). The vacuum pressure of the analysis chamber was $10\times 10^{-9}$ mBar throughout the experiment and a beam size of 400 microns was used. The general spectra were obtained by using a pass energy of 160 eV. The quantitative analysis was performed from high resolution spectra averaged from three points located in different areas on the surface of each of the samples. Once the spectra were obtained, the areas of C 1*s*, N 1*s* and O 1*s* were determined using the software AVANTAGE V5.937 by Thermo Scientific, Waltham, MA, USA.

### 2.6. GAB Parameters

For determining the adsorption properties of unmodified and esterified amaranth starches, samples of 1 g were put into dishes. The dishes were placed into hermetically sealed jars (one dish per jar) at 25 °C, each jar containing one of the following saturated solutions: LiCl, CH_3_CO_2_K, KCl, K_2_CO_3_, Mg(NO_3_)_2_, NaCl, KCl and BaCl_2_. In this way, the water activity $a_{w}$ ranges from 0.11 to 0.94, where $a_{w}=0.11$ corresponds to the jar containing LiCl and $a_{w}=0.94$ corresponds to the jar containing BaCl_2_. Samples were periodically weighed until a constant weight in an analytical balance was reached. Moisture adsorption isotherms were fitted by GAB model, which is described by the formula:

$$M\left(a_{w}\right)=\frac{M_{0}CKa_{w}}{\left(1-Ka_{w}\right)\left(1-Ka_{w}+CKa_{w}\right)}$$

where $M=M\left(a_{w}\right)$ is the moisture content in the sample (g water per 100 g of dry solids) at $a_{w}$, $M_{0}$ is the monolayer moisture content (g water per 100 g of dry solids), *C* is the Guggenheim’s parameter and *K* is a dimensionless parameter. The GAB model is applicable in the range of $0.1\leq a_{w}\leq 0.9$. The GAB model introduces a second well-differentiated adsorption stage resulting from the addition of an extra degree of freedom (the *K* parameter) in its formula. The GAB model usually fits well to the experimental data and is used to predict the monolayer moisture content.

The fitting of isotherm models to the experimental data was evaluated by means of the mean absolute percentage error that is defined as follows

$$P=\frac{100}{N}\sum_{i=1}^{N}\left|\frac{Y_{i}-Y_{i}^{\prime }}{Y_{i}}\right|$$

where *N* is the number of data points, $Y_{i}$ denotes the experimental data and $Y_{i}^{\prime }$ is the forecast value obtained from the model. The mean absolute percentage error has been widely used as a measure of the forecast accuracy of models, and it has been recommended in most textbooks for its reliability [26].

### 2.7. X-ray Diffraction

The X-ray diffraction patterns (XRD) of unmodified and esterified amaranth starches were obtained on a Rigaku Miniflex 600 (Rigaku Denki Co. Ltd, Akishima-shi, Tokyo, Japan) diffraction instrument operating at 40 kV and 15 mA with CuK radiation wavelength of λ = 1.54 Å. The sweeping angle ranged from 2° to 60° on a scale with a step size of 0.01 and 0.03°/min. The relative crystallinity (denoted by $R_{c}$) of starch granules was calculated as the ratio of the crystalline area to the total area under the major diffraction peaks [27]. The software used to analyze the spectrum was MDI Jade 5.0 (free version), from Materials Data JADE, Livermore, CA, USA.

### 2.8. Thermal Properties of Starches

Thermal properties of starches were determined in a DSC (822e, birefrigerated, Mettler Toledo Lab Plant, Huddersfield, UK). The equipment was calibrated with indium, which has a melting point of 156.4 °C and an enthalpy of ΔH = 6.8 cal/g. For each sample, approximately 3 mg (dry basis) were directly weighed into aluminum trays, and deionized water was added in a relation of 2:1 of starch weight. The pan was hermetically sealed to prevent water loss during DSC scanning and equilibrated for 3 h. After that, the pan was first cooled from room temperature to −90 °C at 10 °C/min, and then heated at the same rate up to 80 °C. During the heating, several parameters were determined: apparent glass transition temperature ($T_{g,ice}^{\prime }$), onset ($T_{o,ice}$) and peak ($T_{p,ice}$) temperatures of ice melting and the enthalpy ($\Delta H_{m,ice}$) of native and esterified amaranth starches. The amount of freezable water ($W_{f}$) was calculated according to Ostrowska-Czubenko et al. [28]. The parameter $W_{f}$ defined as g of water/g of dry polymer was calculated after integration of the melting endotherm, by assuming that the melting enthalpy for both the freezing free water ($W_{ff}$) and the freezing bound water ($W_{fb}$) are the same as those of the bulk water ($\Delta H_{0}$ = 334 J/g). The amount of freezable water was calculated according to the formula

$$W_{f}=\frac{\Delta H_{m}}{\Delta H_{0}}$$

where $\Delta H_{m}$ (J/g) is the melting enthalpy for freezable water in starch samples obtained from the DSC thermogram and $\Delta H_{0}$ is the melting enthalpy of pure water. The onset ($T_{o,gel}$) and peak ($T_{p,gel}$) temperatures of gelatinization and the enthalpy ($\Delta H_{gel}$) in J/g of native and derivative starches were also determined. After the analysis, the pan was stored at 4 °C for 7, 14 and 28 days, and the recrystallization (retrogradation) was measured under the same conditions.

### 2.9. Statistical Analysis

Experiments were performed in triplicate. The software SigmaPlot Version 11.0 (Systat Software Inc., San José, CA, USA) was used to conduct an analysis of variance (ANOVA) in order to determine differences between means of treatments. Treatment means were considered significantly different at p<0.05 using pair-wise multiple comparison procedures (Holm–Sidak method).

## 3. Results

### 3.1. Degree of Substitution and Surface Characterization

The degree of substitution (DS) refers to the average number of the hydroxyl groups substituted per anhydrous glucose unit (AGU) in starch. The maximum possible value of DS is 3.0, according to the three free hydroxyl groups at positions C-2, C-3 and C-6 in a pyranose ring of starch [21,29].

**Figure 1:**
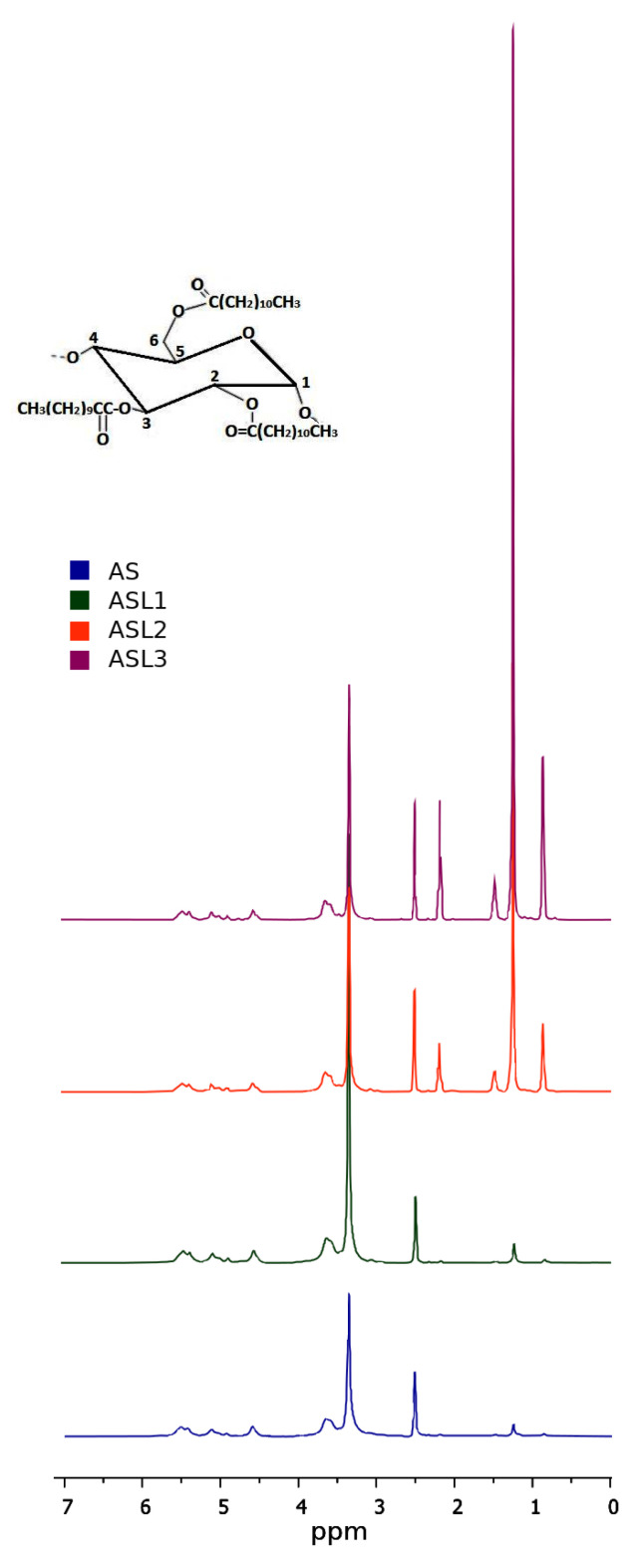
^1^H NMR spectra of native (AS) and modified amaranth starches (ASL1, ASL2 and ASL3).

Figure 1 shows the ^1^H RMN spectra of amaranth starch and its derivatives. All samples exhibit signals of the four protons in AGU at 5.50 (OH-C3), 5.40 (OH-C2), 4.58 (OH-C6) and 4.90 (OH-C4) [30]. For AGU in starch, there exist three hydroxyl groups linked to C-2, C-3 and C-6, and all of them are available to react with lauroyl chloride. After lauroylation, starch gives rise to three new signals identified as acyl protons attached to O-6, O-3 and O-2 on AGU. The signal at 2.2 ppm is related to the methylene group (–CH_2_–) beside the carbonyl group, and the one at 1.5 ppm is the methylene group directly before it, and the rest of the methylene groups show a signal at 1.25 ppm [21]. Table 1 shows the values of DS for the esterified starches, calculated by ^1^H NMR. It is observed that DS increased as the level of modification increased. The yield of esterification was 15%, 98.8% and 92.8% for ASL1, ASL2 and ASL3, respectively. Therefore, the optimal molar ratio was 5 mL of lauroyl chloride (20.8 mmol) for 4 g of starch (25 mmol AGU).

**Figure 2:**
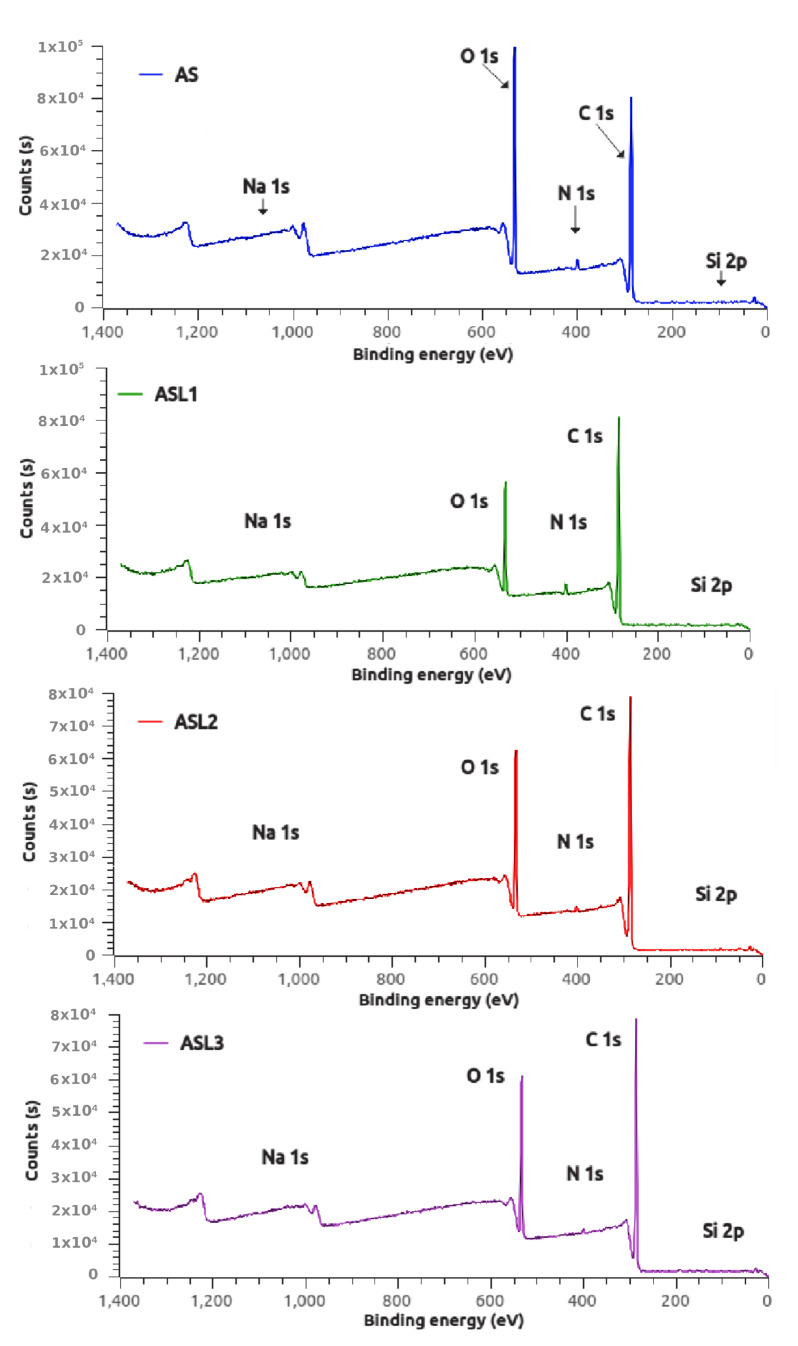
X-ray photoelectron spectroscopy of native (AS) and modified amaranth starches (ASL1, ASL2 and ASL3).

Table 1 presents the XPS results of the elemental composition of approximately 20 Å depth [31] of unmodified (AS) and esterified amaranth starch granules (ASL1, ASL2 and ASL3). The following peaks were detected at different binding energies by the XPS survey scans shown in Figure 2. The peak observed at the binding energy of 101 eV is assigned to Si from silicon oils (C_2_H_6_SiO), which is a contaminant commonly found at the surfaces of some materials exposed to ambient air [32]. The peak observed at the binding energy of 284.6 eV is assigned to C, which makes single bonds with C (C–C) or H (C–H) in lauric acid [33], anhydrous glucose ring or hydrocarbons (CH_2_). The peak observed at the binding energy of 399.7 eV is assigned to uncharged N in amine (C–NH_2_) or in amide groups, which are present in amaranth proteins [23]. The peak observed at the binding energy of 532.6 eV is assigned to O, which makes single bonds with C in alcohol (C–OH) and acetal (RO–C–OR) and hemiacetal (RO–C–OH) in polysaccharides [34]. The peak observed at the binding energy of 1073.9 eV is assigned to Na^+^, attributed to the binding affinity with carboxylate ions [32].

According to Table 1, there were changes in the relative elemental composition of starches. S reduction in the O/C ratio and a corresponding increase in the contribution of C1 carbons (C–C and C–H) for the esterified samples (ASL1, ASL2 and ASL3) were observed. These results are associated with the increase of fatty acid content on the surface of starch derivatives and confirm the esterification of the surface of amaranth starch. To determine the molecular composition of the unmodified and esterified starches, a linear system of equations was formulated from the data in Table 1

$$\begin{array}{cc} 12s+h+6g+4.8p+12a & =n_{C}\\ 15s+5g+1.9p+a & =n_{O}\\ a & =n_{\mathrm{Na}}\\ 1.3p & =n_{N}\\ s & =n_{S} \end{array}$$

where $n_{X}$ represents the fraction ×100 of an X element experimentally observed, *g* is the number of glucose residues, *a* is the number of lauroyl groups, *p* is the number of amino acid residues, *s* is the number of silicon oil molecules and *h* is the number of hydrocarbon groups.

The solutions of the system of equations corresponding to native and esterified starches are shown in Table 1. The DS values on the surface of starch derivatives can be calculated by XPS as the ratio of a/g. For ASL1, the DS thus calculated was higher (0.08) than the value determined from ^1^H NMR (0.06). This suggests that the surface of ASL1 is more enriched with fatty acyl chains than the bulk. However, as DS increases (ASL2 and ASL3), the lauroyl chloride could have diffused more inside the granule and could have modified the crystalline structure of starches (see Section 3.3). The surface of the starch granules in the ASL3 sample was not modified to a greater extent as the amount of reagent increased. It seems to be a limited esterification on the surface of the granule since the ratio O/C remained constant in ASL2 and ASL3.

### 3.2. Adsorption Isotherm Studies

**Figure 3:**
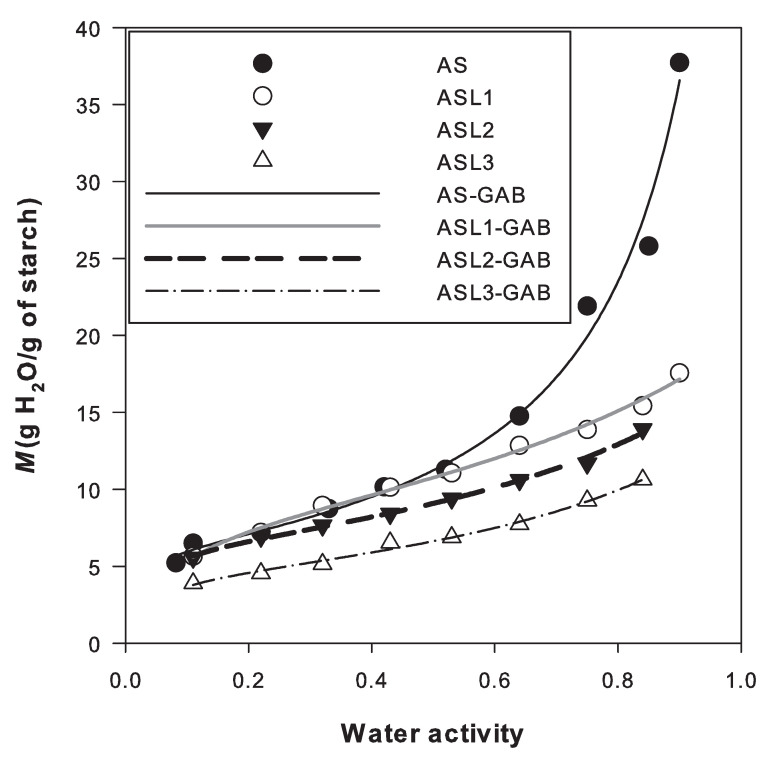
Water adsorption isotherms of native (AS) and modified amaranth starches (ASL1, ASL2 and ASL3) prepared at 25 °C. The solid and dashed lines show the fitting of the GAB model.

Experimental data of equilibrium moisture content for native amaranth and lauroylated starches were fitted by the GAB model (see Figure 3). The parameter *K* determines the rate of growth of the isotherm curve at the higher values of $a_{w}$. The parameter *C* is used for classifying the adsorption isotherms. The parameter $M_{0}$ represents the saturation of polar groups corresponding to the water adsorbed at the most active sites of food [35]. The GAB model is well posed if parameters *K* and *C* are in the ranges of 0.24<K≤1 and 5.76≤C<∞ [35]. The GAB parameters for the different starches are shown in Table 2. It is observed that these parameters agree with the recommended values for having a good fitting. From the values of parameter *C*, it follows that the adsorption isotherms can be classified as of Type II (C>2) since the curves have the typical sigmoidal shape with one inflection point. According to the table, the value of *K* decreases once the amaranth starch was esterified. Such decrease can be attributed to the reduction of moisture adsorption. Recall that moisture adsorption depends on the chemical structure of α-D-glucose units, being the hydroxyl groups of starch responsible for its hydrophilic character. The reduction of free hydrophilic functional (–OH) groups and the esterification with lauroyl chloride reduced the moisture adsorption, as shown in Figure 3. A remarkable reduction of ≈40% of moisture adsorption in ASL3 is observed at values of $a_{w}>0.5$, compared to native amaranth starch.

Regarding the parameter $M_{0}$, it is used to define the physicochemical stability of foods because it has a direct influence on the lipid oxidation, enzyme activity, non-enzymatic browning, flavour preservation and product structure [36]. According to Table 2, a slight increase in $M_{0}$ was observed in ASL1 after esterification, which can be attributed to the alkaline hydrolysis of the starch subjected to 0.1M NaOH before the esterification reaction. By SEM, a little erosion on the surface of lauroylated amaranth starch with NaOH at the same molarity can be observed [23], while Namazi et al. [21] reported a granular structure of lauroylated potato starch completely destroyed when 0.25 M NaOH was used.

A notable decrease in $M_{0}$ was observed in ASL2 and ASL3 with respect to native amaranth starch (AS). This result is in agreement with the increasingly hydrophobic character induced by the DS discussed in Section 3.1, and was also observed by Cova et al. [37] in the esterification of cassava starch with octenyl succinic anhydride (n-OSA). Cova et al. [37] reported the same value of $M_{0}=6.37$ for succinylated cassava starch at lower DS (0.024) than the value obtained here for ASL2 (DS = 0.82). The differences can be attributed to the molecular structure of the employed reagent. The values of $M_{0}$ expressed in mol of water per mol of anhydrous glucose monomer shown in Table 2 are 0.63, 0.75, 0.57 and 0.41 for AS, ASL1, ASL2 and ASL3, respectively. The number of polar groups decreased as the DS increased.

### 3.3. Relative Crystallinity

**Figure 4:**
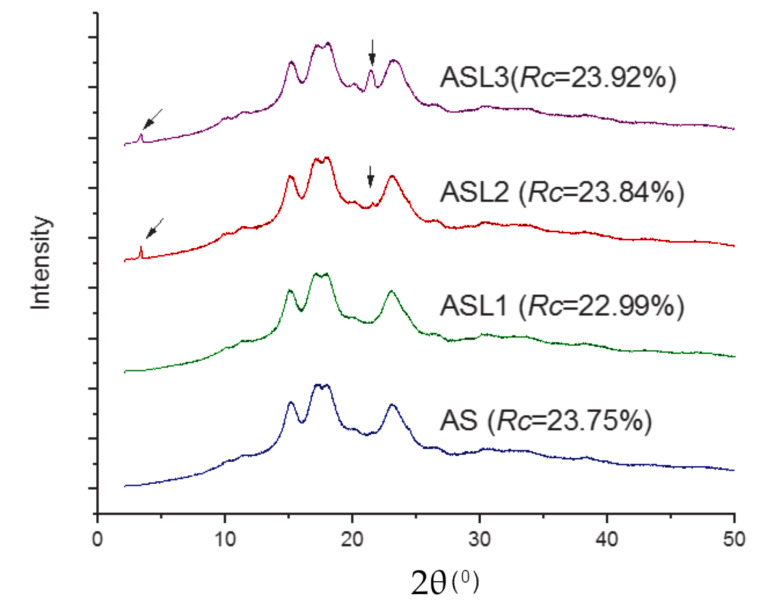
Diffraction patterns and relative crystallinity of native (AS) and modified amaranth starch derivatives (ASL1, ASL2 and ASL3).

The X-ray diffraction (XRD) pattern of the amaranth starch (AS) and amaranth starch laurates (ASL1, ASL2 and ASL3) are shown in Figure 4. AS possessed an A-type X-ray diffraction pattern, which is characterized by intense peaks at 2θ=15° and 23°, and a double peak at 2θ=17° and 18° [38]. The esterification with lauroyl chloride at molar ratio L/AGU of 0.42 (ASL1) had no effect on the XRD pattern, but, at the molar ratios of esterification of 0.83 (ASL2) and 1.25 (ASL3), the peak at 2θ=20° was intensified, and two new peaks at 2θ=3.4° and 21.6° were observed. These peaks were intensified as the DS increased. Similarly, Winkler et al. [39] examined XRD patterns of high amylose maize starch esterified with different long chain fatty acids and two new peaks were detected, namely: at 2θ=5° and 19.6° for starch hexanoate; at 2θ=7° and 19.6° for starch palmitate; and at 2θ=3.2° and 19.6° for starch laurate. Chen et al. [40] reported three peaks at 2θ=7°, 13°, 20° for starch-lauric acid complex formed by microfluidization. These peaks were attributed to $V_{6I}$-type inclusion conformations with six glucose units per turn. The intensity of the characteristic peak of amylose–lipid complex at 2θ≈20° depends on the amylose content. The amylose content of amaranth starch is 22.3±0.3 [23]. The presence of the peak at 21.6° in ASL2 and ASL3 is attributed to the self-association of fatty acids. Marinopoulou et al. [41] studied the formation of high amylose starch-fatty acids complexes with myristic, palmitic and stearic acids and reported the presence of a peak at 2θ≈22°. This peak is allied to the fatty acid crystals entrapped in the helices of the crystallized complexes. The changes in the diffraction patterns are consistent with the relative crystallinity of native and amaranth starch derivatives observed in Figure 4. The increase of crystallinity in the samples of ASL2 and ASL3 is mainly due to the amylose-lauric acid complex and the content of free lipids. ASL3 was the highest, followed by ASL2, which agrees with the gelatinization properties of starches discussed in Section 3.4.

In a similar study, Winkler et al. [39] investigated the thermal and crystallinity properties of fatty acids starch esters derived from high amylose maize starch. The starch structure was disrupted with DMSO and proceeded with the esterification. They reported the X-ray diffraction patters of starch esters with a DS > 2. An effect was detected about 2θ=4°; in the case of starch hexanoate, a peak appeared at 2θ=5°; for starch laurate, at 2θ=3.2°; and for starch palmitate, at 2θ=2.7°. This peak shift downward with increasing the length of fatty acid ester group. In our study, we had a similar peak as the one presented with starch laurate, even though this peak was less intense as the one describe in the related study, which might be attributed to a lower amount of amylose presented in the amaranth starch compared with the high amylose maize starch and other reason could be that in our investigation granule structure were preserved during esterification.

### 3.4. Thermal Characterization

The changes in the ice melting enthalpies of the samples are shown in Table 3. During the cooling of the samples, a mixture of ice and liquid water was present [42]. Therefore, during heating, $T_{g}^{\prime }$ is detected before $\Delta H_{m,ice}$ of ice melting. Table 3 shows that $T_{g}^{\prime }$ decreased and $\Delta H_{m,ice}$ increased as the DS increased. These observations correspond to an increase in the proportion of freezable water ($W_{f}$) from 0.41 to 0.47 (g of water/ g of sample). The $W_{f}$ includes bound and free water; in other words, the substitution of free hydrophilic functionals (–OH) by fatty acyl chains increases $W_{f}$.

After the ice melting, samples of amaranth starch showed two endothermic peaks. The endothermic peak about 38–45 °C was due to the melting of the free fatty acid and the endothermic peak about 145–160 °C was due to the dissociation of starches-fatty acid complexes. The gelatinization and retrogradation parameters of starches are shown in Table 4. The gelatinization properties of native and esterified amaranth starches were compared in fresh samples. Temperatures $T_{0}$ and $T_{p}$ of gelatinization at high levels of modification in ASL2 and ASL3 were statistically (p<0.05) higher than those of native starch (AS), while at the lower level of modification (ASL1) slight changes were observed. The $\Delta H_{m}$ increased drastically in ASL2 and ASL3, which means that more energy was required for the gelatinization. This behavior was attributed to the addition of covalent bonds into the starch molecules [43] and the energy associated with disordering crystallized complexes.

The retrogradation of starches at 4 °C was analyzed for 28 days, and the results are shown in Table 4. Starch retrogradation is influenced by botanical source, molecular composition and structure, temperature fluctuation and concentration of starch gel [44]. After seven days of storage for AS and ASL1, temperatures $T_{0}$ and $T_{p}$ increased and ΔH of retrogradation decreased significantly (p<0.05). No significant differences were observed in ASL2 and ASL3 samples after 28 days of storage. The lower tendency to retrogradation is attributed to the esterification of starch. Similarly, Abiddin et al. [45] observed a lower tendency to retrogradation in *n*-OSA starch.

## 4. Conclusions

The adsorption isotherms of native and lauroylated amaranth starches were well fitted by the GAB model, and the monolayer moisture content derived from the model is an adequate parameter to determine the affinity of water molecules to the starch’s surface. The esterification of amaranth starch with lauroyl chloride increased its hydrophobicity given that lauroylated starches decrease their equilibrium moisture content as the degree of substitution increases. As a result of esterification, an increase in freezable water was observed as the degree of substitution increased due to the reduction of the hydrophilicity of the starches.

The esterification with lauroyl chloride modified the A-type X-ray diffraction pattern of amaranth starch. When DS ≤1.16, the formation of V-type inclusion complexes and self-associated fatty acids during lauroylation were observed. These results give rise to an increase of the thermal properties ($T_{0}$, $T_{p}$ and $\Delta H_{m,ice}$) of lauroylated starches, as well as the prevention of retrogradation.

Based on the results obtained in this investigation, we have the hypothesis that lauroylated amaranth starch may have the potential to be used as a food-grade emulsifier, as well as for the preparation of biodegradable plastic films due to its amphiphilic properties. Of course, such hypothesis should be rigorously proved in the laboratory, which establishes a natural path to follow in this research.

## Figures and Tables

**Table 1:** DS results determined by NMR, XPS results and molecular composition of surfaces of unmodified amaranth starch (AS), and amaranth starch derivatives (ASL1, ASL2 and ASL3).

Sample	DS	O/C	a/g	XPS (%)					Molecular Composition ${}^{1}$				
				**C**	**O**	**Na**	**N**	**Si**	g	a	h	p	s
AS	0	0.50	0	65.5	32.7	0	1.3	0.5	6.06	0	23.34	1	0.5
ASL1	0.06	0.27	0.08	77.1	20.7	0.3	1.5	0.4	3.81	0.3	47.50	0.5	0.4
ASL2	0.82	0.30	0.26	73.6	22.2	0.9	1.0	2.3	3.51	0.9	33.44	0.77	2.3
ASL3	1.16	0.30	1.01	73.1	21.8	3.3	1.0	0.8	3.25	3.3	8.71	0.77	0.8

${}^{1}$*g* = glucose residue (C${}_{6}$O${}_{5}$H${}_{10}$); *a* = lauroyl (C${}_{12}$O${}_{1}$H${}_{23}$Na); *h* = hydrocarbon ($CH_{2}$); *p* = protein (C${}_{4.8}$O${}_{1.9}$N${}_{1.3}$H${}_{7.7}$) [32]; *s* = silicon oil (C${}_{2}$H${}_{6}$SiO).

**Table 2:** Parameters of GAB model for modeling the moisture adsorption isotherms of starches at 25 °C.

GAB Model	Starches			
	**AS**	**ASL1**	**ASL2**	**ASL3**
*C*	30.82	24.23	63.08	40.98
*K*	0.893	0.590	0.642	0.691
$M_{0}$ (g H${}_{2}$O/g of starch)	7.06	8.335	6.374	4.539
*P*(%)	8.72	25.65	14.19	14.76
$R^{2}$	0.995	0.994	0.995	0.992

**Table 3:** Effect of the lauroylation on the ice melting properties determined by DSC.

Analysis	Samples ${}^{1}$			
	**AS**	**ASL1**	**ASL2**	**ASL3**
$T_{g}^{\prime }$ (°C)	−7.73 ± 0.46 ${}^{a}$	−8.112 ± 0.12 ${}^{b}$	−9.84± 0.08 ${}^{c}$	−11.18± 0.08 ${}^{d}$
$T_{0}$ (°C)	−2.67 ± 0.15 ${}^{a}$	−2.38 ± 0.58 ${}^{a}$	−2.69 ± 0.14 ${}^{a}$	−1.96 ± 0.28 ${}^{a}$
$T_{p}$ (°C)	0.49 ± 0.77 ${}^{a}$	0.51 ± 0.36 ${}^{a}$	0.28 ± 0.37 ${}^{a}$	0.22 ± 0.58 ${}^{a}$
$\Delta H_{m,ice}\left(J/g\right)$	137.17 ± 5.46 ${}^{ae}$	144.09 ± 3.09 ${}^{ce}$	145.71 ± 2.37 ${}^{de}$	155.82 ± 2.94 ${}^{b}$
$W_{f}\left(g/g\right)$	0.41 ± 0.02 ${}^{a}$	0.43 ± 0.01 ${}^{a}$	0.44 ± 0.01 ${}^{a}$	0.47 ± 0.00 ${}^{b}$

${}^{1}$$T_{0}$, onset temperature; $T_{p}$, peak temperature; $\Delta H_{m,ice}$, ice melting enthalpy. Different superscripts in the same row indicate significant differences (p<0.05) (n=3).

**Table 4:** Thermal characteristics of fresh starches (AS, ASL1, ASL2 and ASL3) and retrograded starches at different storage durations (r7, r14 and r28).

Samples	$T_{0}$(°C)	$T_{p}$(°C)	$\Delta H_{m}$(J/g)${}^{1}$
AS	38.41 ± 0.42 ${}^{a,A}$	40.19 ± 0.52 ${}^{a,A}$	3.11 ± 0.28 ${}^{a,A}$
AS-r7	42.75 ± 0.99 ${}^{b}$	52.03 ± 1.592 ${}^{b}$	1.70 ± 0.34 ${}^{b}$
AS-r14	42.06 ± 0.63 ${}^{b}$	52.44 ± 0.42 ${}^{b}$	1.63 ± 0.08 ${}^{b}$
AS-r28	43.06 ± 0.12	53.19 ± 0.49 ${}^{b}$	1.54 ± 0.02 ${}^{b}$
ASL1	38.88 ± 1.06 ${}^{a,AC}$	40.18 ± 0.46 ${}^{a,A}$	1.04 ± 0.25 ${}^{a,A}$
ASL1-r7	43.74 ± 2.77 ${}^{b}$	51.84 ± 0.92 ${}^{c}$	1.09 ± 0.24 ${}^{a}$
ASL1-r14	40.6 ± 0.26 ${}^{b}$	51.49 ± 0.38 ${}^{cb}$	1.06 ± 0.02 ${}^{a}$
ASL1-r28	39.05 ± 0.42 ${}^{ab}$	54.21 ± 0.13 ${}^{b}$	1.01 ± 0.06 ${}^{a}$
ASL2	40.23 ± 0.36 ${}^{a,BC}$	41.87 ± 0.26 ${}^{a,B}$	14.31 ± 0.19 ${}^{a,C}$
ASL2-r7	39.87 ± 0.46 ${}^{a}$	40.97 ± 0.59 ${}^{a}$	13.50 ± 0.27 ${}^{a}$
ASL2-r14	39.78 ± 0.47 ${}^{a}$	41.29 ± 0.28 ${}^{a}$	12.97 ± 0.09 ${}^{b}$
ASL2-r28	39.43 ± 0.56 ${}^{a}$	40.84 ± 0.51 ${}^{a}$	12.99 ± 0.03 ${}^{b}$
ASL3	40.99 ± 0.21 ${}^{a,B}$	42.13 ± 0.45 ${}^{a,B}$	23.67 ± 1.74 ${}^{a,B}$
ASL3-r7	40.53 ± 0.16 ${}^{ac}$	42.176 ± 0.32 ${}^{a}$	21.45 ± 1.16 ${}^{a}$
ASL3-r14	40.26 ± 0.22 ${}^{ac}$	42.003 ± 0.04 ${}^{a}$	21.82 ± 0.92 ${}^{a}$
ASL3-r28	40.61 ± 0.32 ${}^{bc}$	42.18 ± 0.13 ${}^{a}$	22.55 ± 0.84 ${}^{a}$

${}^{1}$$T_{0}$, onset temperature; $T_{p}$ peak temperature; $\Delta H_{m}$, melting enthalpy. Different superscripts in the same column for each sample indicate significant differences (p<0.05). Different capital letters superscripts in the same column for different samples indicate significant differences (p<0.05) (n=3).

## Abbreviations

The following abbreviations are used in this manuscript:

DS	Degree of Substitution
GRAS	Generally Recognized As Safe
GAB	Guggenheim–Anderson de Boer model
XPS	X-ray Photoelectron Spectroscopy
XRD	X-ray Diffraction
DSC	Differential Scanning Calorimetry
AGU	Anhydrous Glucose Unit
AS	Amaranth Starch
ASL	Amaranth Starch Laurate
NMR	Nuclear Magnetic Resonance
